# cGMP kinase: past, presence and future

**DOI:** 10.1186/2050-6511-14-S1-O11

**Published:** 2013-08-29

**Authors:** Franz Hofmann, Florian Loga, Katrin Domes, Enrico Patrucco, Jörg W Wegener

**Affiliations:** 1 FOR 923, Institut für Pharmakologie und Toxikologie, Technische Universität München, Germany

## 

Signaling via cGMP-dependent protein kinase is the major pathway of the NO/cGMP cascade in vascular smooth muscle (SM), heart, CNS, and other cells. cGMP-dependent protein kinases (cGK) are serine/threonine kinases that are widely distributed in eukaryotes. Two genes – *prkg1* and *prkg2* - code for cGKs, namely cGKI and cGKII. In mammals, two isozymes, cGKIα and cGKIβ, are generated from the *prkg1* gene. The cGKI isozymes are prominent in all types of smooth muscle, platelets and specific neuronal areas such as cerebellar Purkinje cells, hippocampal neurons, and the lateral amygdala. The cGKII prevails in the secretory epithelium of the small intestine, the juxta-glomerular cells, the adrenal cortex, the chondrocytes, and in the nucleus suprachiasmaticus. Both cGKs are major down- stream effectors of many, but not all signalling events of the NO/cGMP and the ANP/BNP/CNP/cGMP pathways. cGKI relaxes smooth muscle tone and prevents platelet aggregation, whereas cGKII inhibits renin secretion, chloride/water secretion in the small intestine, the resetting of the clock during early night and endochondreal bone growth. cGKs are involved in cardiovascular and non-cardiovascular processes and are modulators of cell growth and many other functions.

Recent evidence suggest that vascular smooth muscle relaxation is not affected by cGK-dependent phosphorylation of smooth muscle expressed TRPC channels, but by cGK-dependent inhibition of TRPC-6 in endothelial cells and attenuation of endothelial release of smooth muscle relaxing factors. Angiotensin increases blood pressure and induces cardiac hypertrophy. Hypertrophy is reduced in sildenafil treated wild type mice but not in beta rescue mice. This suggests that the sildenafil effects are mediated by inhibition of a cGMP hydrolysing phosphodiesterase. Restricted evidence supports the notion that sildenafil affects cGMP levels in cardiomyofibroblasts and/or in cardiac endothelial cells.

It will be shown that cGK are essential modulators of many regulatory processes.

